# Increases in a Pro-inflammatory Chemokine, MCP-1, Are Related to Decreases in Memory Over Time

**DOI:** 10.3389/fnagi.2019.00025

**Published:** 2019-02-13

**Authors:** Brianne M. Bettcher, John Neuhaus, Matthew J. Wynn, Fanny M. Elahi, Kaitlin B. Casaletto, Rowan Saloner, Ryan Fitch, Anna Karydas, Joel H. Kramer

**Affiliations:** ^1^Rocky Mountain Alzheimer’s Disease Center, Departments of Neurosurgery and Neurology, University of Colorado Anschutz Medical Campus, Aurora, CO, United States; ^2^Department of Epidemiology and Biostatistics, University of California, San Francisco, San Francisco, CA, United States; ^3^Department of Neurology, Memory and Aging Center, University of California, San Francisco, San Francisco, CA, United States; ^4^Department of Psychological and Brain Sciences, Washington University in St. Louis, St. Louis, MO, United States; ^5^Department of Psychiatry, San Diego State University/University of California San Diego Joint Doctoral Program in Clinical Psychology, San Diego, CA, United States; ^6^HIV Neurobehavioral Research Program, Department of Psychiatry, University of California, San Diego, San Diego, CA, United States

**Keywords:** chemokines, inflammation, CCL2, memory, longitudinal, cognitive aging

## Abstract

**Objective**: To determine the longitudinal relationship between monocyte chemotactic protein 1 (MCP-1)/CCL2 and memory function in older adults.

**Methods**: We examined longitudinal plasma MCP-1/CCL2 levels and a longitudinal verbal memory measure (CVLT-II 20’ recall) in a sample of 399 asymptomatic older adults (mean age = 72.1). Total visits ranged from 1 to 8, with an average time of 2.1 years between each visit, yielding 932 total observations. In order to isolate change over time, we decomposed MCP-1/CCL2 into subject-specific means and longitudinal deviations from the mean. The decomposed MCP-1/CCL2 variables were entered as predictors in linear mixed effects models, with age at baseline, sex, and education entered as covariates and recall as the longitudinal outcome. In follow-up analyses, we controlled for global cognition and *APOE* genotype, as well as baseline vascular risk factors. We also examined the specificity of findings by examining the longitudinal association between the MCP-1/CCL2 variables and non-memory cognitive tests.

**Results**: Within-subject increases in MCP-1/CCL2 levels were associated with decreases in delayed recall (*t* = −2.65; *p* = 0.01) over time. Results were independent of global cognitive function and APOE status (*t* = −2.30, *p* = 0.02), and effects remained when controlling for baseline vascular risk factors (*t* = −1.92, *p* = 0.05). No associations were noted between within-subject increases in MCP-1/CCL2 levels and other cognitive domains.

**Conclusions**: In an asymptomatic aging adult cohort, longitudinal increases in MCP-1/CCL2 levels were associated with longitudinal decline in memory. Results suggest that “healthy aging” is typified by early remodeling of the immune system, and that the chemokine, MCP-1/CCL2, may be associated with negative memory outcomes.

## Introduction

Dysregulation of immune cascades is considered to be a core component of aging and has been linked with negative pathological outcomes. The impact of the peripheral immune system on cognitive aging outcomes has been controversial, however, with higher levels of circulating inflammatory markers often being conceptualized as non-specific, downstream effects of aging with unclear significance to the central nervous system (CNS; Galasko and Montine, 2010; Galasko and Golde, 2013). Recent studies suggest, however, that the peripheral milieu actively communicates with the CNS, such that blood-borne, circulating immune markers may not only accelerate the peripheral aging process (i.e., immunosenescence; Franceschi et al., 2000; Baruch et al., 2013), but may also directly impact the CNS by impeding hippocampal neurogenesis (Villeda et al., 2011) and exacerbating cognitive decline (Cunningham et al., 2009; Westin et al., 2012).

Accumulating evidence indicates that chemokines, in particular, may play a critical role in bi-directional immune communication between the periphery and the CNS. Chemokines are chemoattractant cytokines that mediate inflammatory responses and regulate immune cell trafficking (i.e., migration of monocytes and lymphocytes). Monocyte chemotactic protein-1 (MCP-1/CCL2) was the first discovered human CC family chemokine (chr.17, q11.2; Van Coillie et al., 1999) and has been identified as a key pro-aging immune factor involved in cellular senescence (i.e., the Senescence-Associated Secretory Phenotype; Fumagalli and d’Adda di Fagagna, 2009). Animal studies suggest that blood-borne MCP-1/CCL2 levels are elevated in older mice and young heterochronic parabionts (i.e., a young mouse whose vascular system is joined with an older mouse), are linked with age-related decline in neurogenesis, and are associated with poorer memory function (Villeda et al., 2011; Villeda and Wyss-Coray, 2013); moreover, MCP-1/CCL2 knockout mice evidence increased neurogenesis in the dentate gyrus subsequent to cranial irradiation (Lee et al., 2013).

Human studies of MCP-1/CCL2 levels have extended these findings, and demonstrated that baseline blood MCP-1/CCL2 levels in late life are associated with more pernicious clinical outcomes (Lee et al., 2018). Recent work from our laboratory showed that blood MCP-1/CCL2 levels have selective cognitive and neuroanatomical relevance in both typical aging as well as early stages of Alzheimer’s disease (AD), such that higher MCP-1/CCL2 levels are related to worse episodic memory function and smaller left medial temporal lobes. Moreover, study results suggested that associations between MCP-1/CCL2 and cognition were both *sensitive and specific* to episodic memory, as no effects were observed between the circulating chemokine and other indices of neuropsychological function (Bettcher et al., 2016). We also recently demonstrated that in the context of lower CSF levels of Aβ42 (i.e., reflecting higher brain amyloid load), higher levels of blood MCP-1/CCL2 levels were correlated with higher levels of CSF phosphorylated and total tau in asymptomatic older adults (Bettcher et al., 2018a). Collectively, these results suggest that blood-borne MCP-1/CCL2 levels may reflect early AD-related pathological changes in the CNS and may specifically relate to worse episodic memory outcomes in aging adults.

Although prior studies highlight predominantly a cross-sectional association between peripheral MCP-1/CCL2 levels and negative aging outcomes, no study to date has addressed whether increases in blood MCP-1/CCL2 levels are related to *tandem* declines in episodic memory over the time. Examination of peripheral MCP-1/CCL2 levels and cognitive outcomes in a longitudinal framework is important and necessary to fully explicate the strength and directionality of associations between circulating inflammation and late life decline. Moreover, delineation of these longitudinal relationships is critical to our understanding of the neurobiological role of peripheral chemokine markers on aging outcomes, and may further explicate the dynamic associations between inflammation and cognitive decline over time (Bettcher and Kramer, 2014). To address this significant gap in the literature, the overarching goal of this study is to assess the longitudinal relationship between MCP-1/CCL2 and memory function in a cohort of asymptomatic older adults. We hypothesized that increases in MCP-1/CCL2 levels would be an independent predictor of memory decline, even after accounting for mean MCP-1/CCL2 levels and global cognitive functions.

## Materials and Methods

### Participants

A sample of 399 clinically healthy, community dwelling older adult participants was obtained from the University of California, San Francisco (UCSF) Memory and Aging Center database based on the availability of memory testing and stored blood specimens, the latter of which was acquired within 120 days of their memory assessment. The sample cohort was assembled from several longitudinal umbrella studies on healthy aging and cognition (i.e., NIH Aging and Cognition study; Larry L. Hillblom foundation study; NIH Chronic Inflammation study), and were between the ages of 50 and 99 years. In addition to measures of episodic memory and global cognitive function, participants also underwent brief cognitive testing that included measures of processing speed, executive functions, language, and visuospatial function. These measures were included in exploratory analyses (see below) to evaluate the specificity of our findings.

In terms of inclusion/exclusion criteria of these umbrella studies, all participants were reviewed in a screening visit, which entailed an informant interview, neurological examination, and cognitive testing. Inclusion as a “neurologically healthy” participant was based on several criteria, including a Mini-Mental State Exam score of ≥26, Clinical Dementia Rating score of 0, and no subject or informant report of significant cognitive decline during the previous year. Participants were excluded if they had a major psychiatric disorder, neurological condition affecting cognition (e.g., Parkinson’s disease, epilepsy; large vessel infarct), dementia or mild cognitive impairment, substance abuse, significant systemic medical illness (e.g., cancer; renal failure), current medications likely to affect CNS functions (e.g., long-active benzodiazepines), significant sensory or motor deficits that would interfere with cognitive testing, current depression (Geriatric Depression Scale Score greater than 15 of 30; Yesavage et al., 1982) or insulin-dependent diabetes.

For the purposes of the current study, participants were included in the data analysis if they were considered healthy [i.e., no diagnosis of mild cognitive impairment (MCI), AD dementia, or any other neurodegenerative phenotype] at their baseline visit^1^. All participants were reviewed at a case conference with a board-certified neuropsychologist (JK) and neurologist. *All longitudinal memory and blood data* *available* for each participant were included in analyses. As noted, data were culled from numerous healthy aging studies, thus longitudinal data for each participant may include data from a single study or multiple studies over time. As such, longitudinal follow-up times for each participant varied (see “Results” section for number of follow-up visits).

Primary demographic and cognitive variables, as well as information regarding the number of observations for each participant are reported in Table 1. The study was approved by the UCSF committee on human research, and all subjects provided written, IRB-approved informed consent before participating and approved the use of their blood samples and data for the study.

**Table 1:** Baseline characteristics of sample and visit summary.

	Mean (SD)
**Demographics**	
Baseline age	72.1 (7.4)
Sex (% female)	55.6%
Education	17.5 (2.0)
*APOE* (at least 1 E4 allele)	24%
	(82 of 337 available)
**Baseline cognitive testing**	
*Global cognition*	
MMSE (total)	29.1 (1.2)
*Memory functions*	
CVLT-II 20’ delay (total)	11.7 (3.0)
*Executive functions*	
Stroop color naming (total in 60”)	84.6 (15.2)
Stroop interference (total in 60”)	49.1 (10.9)
Modified trail making (time, in seconds)	31.2 (18.2)
*Language functions*	
Category fluency (total in 60”)	22.4 (5.2)
*Visuospatial functions*	
VOSP number location (total)	9.0 (1.2)
	**Number of observations**
**Visits with blood MCP-1/CCL2 and memory testing**	
1	399
2	234
3	149
4	80
5	43
6	20
7	5
8	2

### Longitudinal Verbal Episodic Memory Assessment

Our primary outcome variable was longitudinal episodic memory and was selected due to its consistent and robust associations with early stages of AD (Kramer et al., 2003; Ewers et al., 2012; Dickerson and Wolk, 2011) as well as its sensitivity to immune dysregulation (Lin et al., 2009; Bettcher et al., 2012, 2016). Episodic memory was assessed longitudinally with a list-learning measure of verbal memory, the California Verbal Learning Test-II (Delis et al., 2000). For data reduction purposes, 20-min delayed free recall was used as the primary longitudinal variable of interest.

### Longitudinal Laboratory Measurement of MCP-1/CCL2

After collection, each blood sample was centrifuged at 2,000× *g* for 15 min at 4°C with the resultant plasma divided into 500 μL aliquots and stored at −80°C. All plasma assays were conducted following the manufacturer’s protocol for Human Chemokine Panel 1 V-PLEX Plus kit (Meso Scale Diagnostics, Rockville, MD, USA). We elected to use V-PLEX kits given their lot-to-lot consistency for longitudinal analyses (i.e., reducing batch effects). Each array was scanned using a MESO QuickPlex SQ 120. Manufacturer supplied software (Discover Workbench 4.0) was used to quantify the concentration of MCP-1 based on sample dilution and relative to the supplied in-assay standard curve. Nominal recovery for control levels remained between 111–120% with a coefficient of variation (CV) <10%. Standard curve CVs for participant sample detection range remained <15% with standard sample recovery at 100% (+/− 5%) across all plates.

Importantly, we elected to employ a hypothesis-based approach and focus on MCP-1/CCL2 for several reasons. First, recent, striking associations between higher MCP-1/CCL2 levels and reduced hippocampal neurogenesis in parabiosis animal models have been reported (Villeda et al., 2011). Second, human studies of aging suggest that MCP-1/CCL2 may be a critical chemokine involved in memory decline in AD (Westin et al., 2012). In addition, our laboratory has shown that MCP-1/CCL2 is not only sensitive to episodic memory dysfunction in aging and AD, but may also be specific to this cognitive domain (Bettcher et al., 2016). Collectively, recent data suggest that MCP-1/CCL2 levels may be a key regulator of memory outcomes in aging adults and may be dysregulated early in pathological aging trajectories. Nonetheless, in order to account for associations between chemokines and cognitive function outside of our primary hypotheses, we also examined other detectable chemokines from the Human Chemokine V-PLEX in exploratory analyses. Chemokines that were detectable for more than 80% of the sample across longitudinal time points included: Eotaxin-1, IP-10, MCP-4, MDC, TARC, and MIP-1β.

### *APOE* Genotyping

*APOE* single nucleotide polymorphism (SNP) genotyping was carried out by real time polymerase chain reaction (rtPCR), on an Applied Biosystems 7900HT Real Time PCR machine using the Taqman SNP Genotyping Assay for rs429358 and rs7412 with identification numbers C___3084793_20 and C____904973_10, respectively (Applied Biosystems, Foster City, CA, USA). The protocol was followed as outlined in the manufacturer’s instructions, and every assay was performed in duplicate.

### Statistical Analysis

Linear mixed effect models using maximum likelihood estimates with random intercepts were conducted. The predictor of interest was longitudinal MCP-1/CCL2 levels; in order to isolate change over time, we decomposed MCP-1/CCL2 into subject-specific means (i.e., between subject factor) and longitudinal deviations from the mean (i.e., within subject factor). To increase the scientific rigor of the analyses, both decomposed factors were simultaneously entered into the regression models as predictors to elucidate whether the change in MCP-1/CCL2 levels vs. average MCP-1/CCL2 levels were associated with longitudinal change in memory function. Age at baseline, sex, and education were entered as nuisance covariates in all models and 20-min delayed free recall on the CVLT-II served as the longitudinal outcome.

In secondary analyses, linear mixed effects models were repeated, but we also controlled for global cognition [Mini Mental State Examination (MMSE)] and APOE genotype. We also examined whether these longitudinal associations were independent of baseline vascular risk factors by including baseline body mass index (BMI) and baseline systolic blood pressure as nuisance covariates in our mixed effects models.

In exploratory analyses, we first evaluated whether the longitudinal associations between peripheral inflammation and memory were specific to MCP-1/CCL2 by analyzing other chemokines in separate models. Specifically, we repeated the longitudinal measurement approach used with MCP-1 (i.e., decomposing chemokine levels into subject-specific means and longitudinal deviations from the mean) with six control chemokines (i.e., Eotaxin-1, IP-10, MCP-4, MDC, TARC, and MIP-1β) in separate models. These exploratory analyses allowed us to examine whether chemokines not included in our *a priori* hypotheses were dynamically related to memory over time. Next, we also examined whether longitudinal changes in MCP-1/CCL2 levels were specific to longitudinal memory performance by including comparator indices of executive functions (i.e., modified trail making test, total time; stroop inhibition, total correct), processing speed (i.e., stroop color naming, total correct), language (i.e., animal fluency; total correct), and visuospatial function (i.e., VOSP number location test; total correct; Warrington and James, 1991) as separate longitudinal outcome variables. Please see prior publications for additional details on this standard battery of cognitive measures (Kramer et al., 2003).

All longitudinal analyses were conducted with SAS 9.4 using the Proc Mixed routine.

## Results

As noted in Table 1, our participant sample was comprised of 399 aging adults whose baseline ages ranged from 51 to 99 (mean = 72 years). Visits ranged from 1 to 8 with an average of 2.1 years between visits (SD = 1.2 years; minimum: 0.5 years; maximum: 7.6 years), yielding 932 total observations. Higher baseline levels of MCP-1/CCL2 were significantly associated with higher age at baseline (*r* = 0.24; *p* < 0.0001). Correlations between higher baseline MCP-1/CCL2 levels and lower baseline delayed recall were notable for small effect sizes (*r* = −0.10; *p* = 0.03).

### Question 1: Are Within-Subject Increases in MCP-1/CCL2 Levels Associated With Declines in Verbal Memory? (Table 2)

We examined the effects of both the within-subject change in MCP-1/CCL2 levels and the average MCP-1/CCL2 levels on longitudinal memory outcomes, controlling for baseline age, sex, and education level. Results showed that within-subject increases in MCP-1/CCL2 levels (unstandardized beta = −0.011, SE = 0.004, *p* = 0.008) were associated with declines in delayed recall performance on memory testing over time. In contrast, when examining the effect of average MCP-1/CCL2 levels, a unit difference in mean values between participants resulted in a non-statistical difference in outcome (unstandardized beta < 0.0001, *p* = 0.841). As shown in Table 2, longitudinal, within-subject increases in MCP-1/CCL2 levels were associated with a 10× greater effect on longitudinal memory outcomes relative to the average MCP-1/CCL2 levels.

**Table 2:** Mixed effect model results: within-subject increases in monocyte chemotactic protein 1 (MCP-1) are related to longitudinal decreases in memory.

Effect	Estimate	SE	95% CI	*T*-value	*P*-value
Intercept	16.603	1.780	13.135 to 20.072	9.50	<0.0001
Baseline age	−0.099	0.020	−0.136 to −0.062	−5.38	<0.0001
Male sex	−1.461	0.272	−1.995 to −0.927	−5.47	<0.0001
Education	0.175	0.066	0.045 to 0.305	2.61	0.01
Mean MCP-1 levels (between subject effect)	0.000	0.007	−0.013 to 0.013	−0.20	0.84
Deviation MCP-1 levels (within-subject effect)	−0.011	0.004	−0.018 to −0.003	−2.65	0.01

Of note, we decomposed MCP-1/CCL2 values based on subject-specific means in order to protect against subject-level confounding (Neuhaus and McCulloch, 2006); however, we also repeated the analyses using *baseline* MCP-1/CCL2 levels as the between subject effect, and deviation from the baseline MCP-1/CCL2 levels as the within-subject effect; no substantive changes in results were noted (baseline MCP-1/CCL2 levels: estimate = −0.0008; *p* = 0.88; change in MCP-1 levels: estimate = −0.010; *p* = 0.007).

### Question 2: Are Within-Subject Increases in MCP-1/CCL2 Levels Associated with Longitudinal Declines in Verbal Memory, After Controlling for Global Cognitive Function and APOE Status?

To determine whether increases in MCP-1/CCL2 levels were related to decline in delayed memory performance, over and above global cognitive performance and *APOE* status, we repeated the previous analyses with APOE status (i.e., presence or absence of E4 allele) and MMSE performance (i.e., total score, time-varying covariate) as nuisance covariates. Increases in MCP-1/CCL2 levels remained an independent predictor of verbal memory decline after accounting for *APOE* status and MMSE changes over time (change in MCP-1/CCL2: estimate = −0.009; SE = 0.004; *t* = −2.30; *p* = 0.02).

### Question 3: Are Within-Subject Increases in MCP-1/CCL2 Levels Associated with Longitudinal Declines in Verbal Memory, After Controlling for Baseline Vascular Risk Factors?

Considering the known association between vascular risk factors and both inflammatory markers and cognitive decline, we included baseline systolic blood pressure (mean = 134.19 mmHg; SD = 16.96) and baseline BMI (mean = 25.53; SD = 3.92) as predictors in the model, in addition to covariates from the previously described analyses (Questions 1 and 2). When controlling for baseline demographics, blood pressure, BMI, as well as *APOE* genotype and MMSE, within-subject increases in MCP-1/CCL2 remained associated with declines in verbal memory performance, although the effect size was notably reduced (change in MCP-1/CCL2: estimate = −0.008; SE = 0.004; *t* = −1.92; *p* = 0.05).

### Exploratory Analysis 1: Are the Effects of Longitudinal Increases in Inflammation on Memory Specific to MCP-1 (Table 3)?

Based on prior animal work and on our group’s previously published work, we focused on MCP-1 as the primary pro-inflammatory chemokine in analyses; however, to determine if the effects of inflammation on memory function were specific to MCP-1, we examined additional chemokines from the same V-PLEX panel in a set of separate linear models. Partially consistent with our hypotheses, within-subject increases in several comparator chemokines (i.e., Eotaxin-1, MCP-4, TARC, IP-10, and MIP-1β) were not related to longitudinal change in verbal memory (see Table 3); however, within-subject increases in MDC (estimate = −0.001; *t* = −2.17; *p* = 0.03) were significantly related to declines in verbal memory.

**Table 3:** Mixed effect model results: increases in comparator chemokines and their association with longitudinal memory.

Effect	Estimate	SE	95% CI	*T*-value	*P*-value
**Eotaxin-1**					
Mean eotaxin-1 levels	0.004	0.003	−0.002 to 0.010	1.44	0.15
Deviation eotaxin-1 levels	0.000	0.002	−0.004 to 0.004	−0.05	0.96
**MCP-4**					
Mean MCP-4 levels	0.005	0.004	−0.002 to 0.012	1.31	0.19
Deviation MCP-4 levels	−0.002	0.002	−0.007 to 0.002	−1.08	0.28
**TARC**					
Mean TARC levels	−0.003	0.002	−0.008 to 0.001	−1.39	0.16
Deviation TARC levels	0.001	0.001	−0.002 to 0.003	0.48	0.63
**IP-10**					
Mean IP-10 levels	0.001	0.001	−0.001 to 0.003	1.12	0.27
Deviation IP-10 levels	0.000	0.001	−0.001 to 0.001	−0.08	0.94
**MIP-1β**					
Mean MIP-1β levels	0.001	0.007	−0.012 to 0.015	0.19	0.85
Deviation MIP-1β levels	−0.011	0.006	−0.023 to 0.002	−1.65	0.09
**MDC**					
Mean MDC levels	0.000	0.001	−0.001 to 0.001	0.91	0.37
Deviation MDC levels	−0.001	0.000	−0.002 to −0.000	−2.17	0.03

### Exploratory Analysis 2: Are the Effects of Longitudinal Increases in MCP-1/CCL2 Levels on Cognition Specific to Episodic Memory?

Although our prior work points to a specific effect of MCP-1/CCL2 levels on episodic memory, we elected to examine whether these findings remain robust in a longitudinal sample. Consistent with our prior cross-sectional study, within-subject increases in MCP-1/CCL2 were not statistically associated with changes in processing speed (stroop color naming: estimate = 0.022; *t* = 1.43; *p* = 0.15; observations = 901), executive functions (Modified Trail Making Time: estimate = 0.026; *t* = 0.93; *p* = 0.35; observations = 903; stroop inhibition: estimate = −0.008; *t* = −0.59: *p* = 0.55; observations = 899), language (animal fluency: estimate = 0.010; *t* = 1.26; *p* = 0.21; observations = 896), or visuospatial function (VOSP number localization: estimate = 0.001; *t* = 0.28; *p* = 0.78; observations = 903).

## Discussion

In this retrospective, longitudinal analysis of an asymptomatic older adult cohort, we found that increases in MCP-1/CCL2 levels were associated with longitudinal decline in verbal episodic memory. Results were independent of *APOE* status and global cognitive function, and modest effects remained when accounting for baseline vascular risk factors. Moreover, longitudinal effects of MCP-1/CCL2 on cognition were specific to episodic memory. Results are consistent with cross-sectional studies linking blood MCP-1/CCL2 levels to worse episodic memory performance (Bettcher et al., 2016), as well as studies indicating that higher baseline MCP-1/CCL2 levels are related to decline in cognitive function over time (Lee et al., 2018). To our knowledge, however, this is the first study to extend findings to a longitudinal framework (i.e., longitudinal predictors; longitudinal outcome). Our results demonstrate that longitudinal increases in blood MCP-1/CCL2 levels are related to *tandem* declines in episodic memory over time in an asymptomatic aging cohort.

The noted association between increases in MCP-1/CCL2 levels and episodic memory decline highlight several important points, as well as raise questions about the role of immune dysregulation in pathological aging trajectories. First, study results suggest that longitudinal associations between MCP-1/CCL2 levels and memory performance are evident in otherwise healthy older adults. Memory scores were not in the impaired range, indicating that even within the context of “normal” memory performance, within-subject decline can be mapped on to increases in circulating MCP-1/CCL2 levels. Identifying dynamic biomarkers in plasma that are sensitive to clinically meaningful outcomes in asymptomatic adults is important for understanding both heterogeneity of aging trajectories and early disease processes. Our results point to an early and cognitively salient role for MCP-1/CCL2 levels in late life outcomes. Study results further support the possibility of a dynamic association between the peripheral immune milieu and CNS. More specifically, study findings suggest that increases in MCP-1/CCL2 levels are stronger predictors of memory decline than either average or baseline MCP-1/CCL2 levels, with change in MCP-1/CCL2 associated with a 10 × greater effect on memory outcomes than average MCP-1/CCL2 levels. From both a clinical and research perspective, this indicates that ascertaining a single time point or even averaging several time points of MCP-1/CCL2 levels may be less informative than examining the longitudinal, within-person change in levels. The temporal evolution of chemokine levels in the context of salient cognitive outcomes is critical to our understanding of how immune dysregulation might map on to—or even herald—clinical decline; while study results do not pinpoint whether immune dysregulation is an early marker of AD pathogenesis, nor do they address causality due to the non-interventional design, they do suggest that *chronic* immune dysregulation may presage clinical symptomology and may track with subtle memory decline in late life.

Results from our study also suggest that the relationships between longitudinal MCP-1/CCL2 levels and memory decline are independent of *APOE* status and global cognitive performance, with modest effects remaining after controlling for baseline vascular risk factors. We focused our analysis on episodic memory decline based on prior work indicating that MCP-1/CCL2 levels may have specific effects on medial temporal lobe structures and memory in both animal models (Villeda and Wyss-Coray, 2013) and human aging studies (Bettcher et al., 2016). Our exploratory analyses provide additional support for the specificity of MCP-1/CCL2 levels on memory performance, as the effects of within-subject increases in MCP-1/CCL2 on cognition were solely noted with the verbal memory measure in our sample. Thus, although we examined longitudinal associations with measures of processing speed, executive functions, language, and visuospatial function, no significant results were observed with other cognitive domains. Overall, our study results indicate that the strength of the association between increases in MCP-1/CCL2 levels and decreases in episodic memory are not substantively impacted by subjects’ overall cognitive status, and that longitudinal associations between MCP-1/CCL2 and cognition may be specific to memory.

It is worth noting, however, that the effect size of longitudinal MCP-1/CCL2 levels on memory were impacted by the inclusion of baseline vascular risk factors. Results remained statistically significant, but the independent association of longitudinal MCP-1/CCL2 levels on episodic memory was modest after accounting for baseline BMI and baseline systolic blood pressure. The intersection of immune biology and vascular pathways has garnered significant attention in the past decade (Yaffe et al., 2004; Hoshi et al., 2005, 2010; Fornage et al., 2008; Giunta, 2008; Hoth et al., 2008; Zhang et al., 2009; Gallacher et al., 2010; Espinola-Klein et al., 2011; Grammas et al., 2011; Grammas, 2011; Bettcher et al., 2014), but remains a poorly understood mechanism for cognitive decline in late life. Inflammation is by definition a vascular process that stimulates changes in vascular permeability and, when dysregulated, can alter endothelial metabolic pathways and induce neurotoxic effects. (Grammas, 2011) Considering that chemokines and vascular risk factors are both linked to the development of MCI and AD (Kivipelto et al., 2001; Schmidt et al., 2002; Gottesman et al., 2017), examining these factors in isolation likely represents an overly simplistic and potentially myopic approach to understanding the role of immune biology in aging outcomes. Our study suggests that whereas peripheral MCP-1/CCL2 levels may be independent contributors to episodic memory decline, disentangling their role from vascular risk factors is still an important, but understudied area of research that has yet to be resolved.

In follow-up analyses, we also examined whether the negative association between increasing inflammation and decreasing verbal memory was driven primarily by changes in MCP-1/CCL2 levels or by more diffuse alterations in chemokine levels in the periphery. The role of chemokines as potent chemotactic factors for monocytes is well documented, and MCP-1/CCL2 in particular has been strongly linked with age-related diseases, including AD (Deshmane et al., 2009; Bettcher et al., 2016, 2018a; Lee et al., 2018). Of the six comparison or “control” chemokines we examined, significant associations were only noted for increasing MDC/CCL22 levels and declining memory performance, and this association was weaker than that of MCP-1/CCL2. The role of MDC levels as potential mediators of cognitive decline has been far less researched in the field of aging and AD, thus it remains unclear if MDC levels will serve as a possible biomarker for cognitive aging. Nonetheless, while our results corroborate and extend prior reports on the role of MCP-1 in cognitive aging, they also raise important questions regarding the contribution of MDC to memory decline and the sensitivity of specific CC family chemokines to aging outcomes.

An additional consideration when interpreting the longitudinal role of peripheral MCP-1/CCL2 levels on cognitive decline is the mechanism by which the periphery might induce changes in the CNS. Increasing evidence from basic science literature points to an alteration in the milieu surrounding the epithelial monolayers of the choroid plexus (CP), which serves as a critical immune nexus between the blood and CSF. (Baruch et al., 2014). Recent reports suggest that the blood-CSF barrier undergoes functional alterations with age, which may be exacerbated in the presence of (Vargas et al., 2010) and possibly preceding (Chalbot et al., 2011) amyloid deposition. The downstream effects of these age- and pathology-related changes in the CP environment include the potential for increased communication between the periphery and CNS (Dá Mesquita et al., 2016). Although *in vivo* methods for assessing the immune milieu surrounding the CP have not been clearly established in humans, it may serve as a future target for understanding the mechanistic role of peripheral immune dysregulation on cognitive decline.

The current study has numerous strengths, including the longitudinal assessment of both MCP-1/CCL2 levels and verbal episodic memory measurements in healthy older adults, as well as the appraisal of *APOE* status and baseline vascular risk factors. In terms of methodological limitations to the study, we elected to focus on healthy older adults who enrolled in several aging studies at UCSF to maximize sample size and number of observations; as such, our participants reflected a convenience sample of community-dwelling, highly educated older adults rather than an epidemiological sample. Sampling bias and generalizability of study findings remains a critical issue in observational research; future studies should examine whether similar findings are noted using community-based sampling methods with demographically diverse populations. An additional limitation to the current study is the lack of baseline amyloid and tau biomarkers. Heterogeneity in “normal” aging trajectories is characterized in part by the presence of pathology—including vascular disease, amyloid deposition and tau pathology (Jack et al., 2013)—which was not assessed in the current study. Considering that elevations in peripheral inflammatory levels may exacerbate, and even precede, markers of AD-related pathology, it will be important to incorporate these indices to gain a better understanding of the temporal properties of immune dysregulation in late life. Finally, our statistic modeling approach included the use of the MMSE as a measure of global cognitive function; we incorporated this assessment given its availability in our dataset as well as its wide use a proxy for global cognition. In a healthy older adult sample, however, the variability in this measure is limited, and is prone to ceiling affects.

In summary, in a sample of asymptomatic older adults, longitudinal increases in MCP-1/CCL2 levels were related to declines in verbal episodic memory over time, and these results were independent of *APOE* status, with modest effects remaining after accounting for baseline vascular risk factors. Exploratory analyses further suggest that the longitudinal association between MCP-1/CCL2 levels and cognition may be specific to memory. Results add to a growing body of literature suggesting that “healthy aging” is typified by remodeling of the immune system, and that the blood-borne chemokine, MCP-1/CCL2, may be an early marker of innate immune dysregulation that is sensitive to negative memory outcomes.

## Data Availability

The datasets for this study will not be made publicly available because the datasets for this manuscript are not publicly available because of HIPAA-related concerns (i.e., information that could compromise the privacy of research participants; ages over 89). Requests to access the datasets via data use agreements should be directed to Joel H. Kramer, PsyD (joel.kramer@ucsf.edu).

## Author Contributions

BB assisted in collection of the data, developed the hypotheses, conducted the statistical analysis, and wrote the manuscript draft. JN assisted with the statistical analyses and critically revised the manuscript. MW, FE, KC, RS, and AK assisted with data collection and critically revised the manuscript. RF processed all blood specimens, conducted analyses of the inflammatory markers and critically revised the manuscript. JK developed the hypotheses, collected the data, and critically revised the manuscript.

## Conflict of Interest Statement

The authors declare that the research was conducted in the absence of any commercial or financial relationships that could be construed as a potential conflict of interest.

## Footnotes

^1^All participants retained a diagnosis of “healthy control” throughout at all time points in the current study; however, 7% (*n* = 27) were later diagnosed with MCI, AD, or subjective cognitive impairment.
